# Infection-Related Hospitalizations After Simultaneous Pancreas-Kidney Transplantation Compared to Kidney Transplantation Alone

**DOI:** 10.3389/ti.2024.12235

**Published:** 2024-02-20

**Authors:** Juulia Grasberger, Fernanda Ortiz, Agneta Ekstrand, Ville Sallinen, Kaisa Ahopelto, Patrik Finne, Mika Gissler, Marko Lempinen, Ilkka Helanterä

**Affiliations:** ^1^ Transplantation and Liver Surgery, Helsinki University Hospital and University of Helsinki, Helsinki, Finland; ^2^ Nephrology, Helsinki University Hospital and University of Helsinki, Helsinki, Finland; ^3^ Department of Knowledge Brokers, Finnish Institute for Health and Welfare, Helsinki, Finland; ^4^ Academic Primary Health Care Centre, Region Stockholm, Stockholm, Sweden; ^5^ Department of Molecular Medicine and Surgery, Karolinska Institutet, Stockholm, Sweden

**Keywords:** kidney transplantation, pancreas transplantation, infection, bacteremia, infection-related hospitalization, complication, survival

## Abstract

The total burden of infections after transplantation has not been compared in detail between recipients of simultaneous pancreas-kidney transplantation (SPK) and kidney transplantation alone (KTA). We compared infection-related hospitalizations and bacteremias after transplantation during 1- and 5-year follow-up among 162 patients undergoing SPK. The control group consisted of 153 type 1 diabetics undergoing KTA with the inclusion criteria of donor and recipient age < 60, and BMI < 30. During the first year, SPK patients had more infection-related hospitalizations (0.54 vs. 0.31 PPY, IRR 1.76, *p* = <0.001) and bacteremias (0.11 vs. 0.01 PPY, IRR 17.12, *p* = <0.001) compared to KTA patients. The first infection-related hospitalizations and bacteremias occurred later during follow-up in KTA patients. SPK was an independent risk factor for infection-related hospitalization and bacteremia during the first year after transplantation, but not during the 5-year follow-up. Patient survival did not differ between groups, however, KTA patients had inferior kidney graft survival. SPK patients are at greater risk for infection-related hospitalizations and bacteremias during the first year after transplantation compared to KTA patients, however, at the end of the follow-up the risk of infection was similar between groups.

## Graphical Abstract

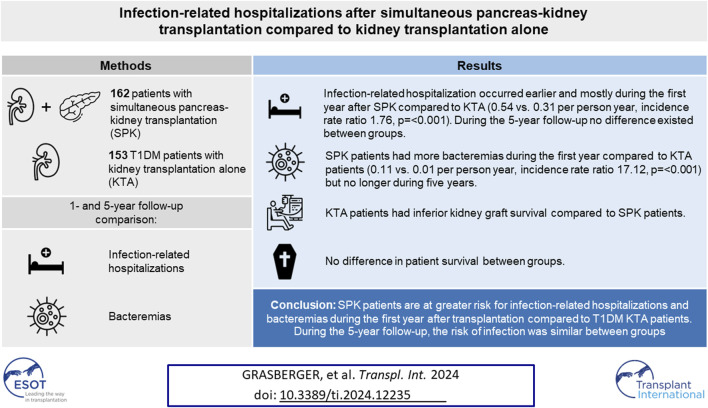

## Introduction

The results of simultaneous pancreas-kidney transplantation (SPK) have improved during the last decades due to advanced surgical techniques and immunosuppressive therapies [1, 2]. Many studies have shown superior patient and kidney graft survival in SPK patients compared to kidney transplantation alone (KTA) [3–8], as well as the reduction of micro- and macrovascular complications of diabetes [9–12]. However, postoperative complications are common in SPK patients with relaparotomy rates reported to range from 23% to as much as 44% [13–15], and the incidence of surgical site infections is among the highest in solid organ transplant (SOT) recipients [16]. Surgical complications cause significant morbidity and may adversely affect the pancreas graft survival [14]. In addition, more intensive immunosuppression, and especially the use of lymphocyte-depleting agents, may predispose SPK patients to increased infections compared to KTA patients.

On the other hand, KTA patients continue to be exposed to the hyperglycemic conditions and studies have shown increased risk of infections [17] and infection-related mortality [18] among patients with diabetes compared to the general population. Also, in a large study comparing infections among kidney transplant recipients, the infection-related mortality was higher for diabetics compared to non-diabetics [19]. Therefore, the ongoing diabetes and hyperglycemia may continue to act as a risk factor for infections in diabetic KTA patients compared to SPK patients who usually achieve normoglycemia after a functional pancreas transplant.

Data about the long-term infectious complications in SPK patients compared to KTA patients are limited and studies comparing the infection burden of specifically diabetic KTA patients with SPK patients do not exist to our knowledge. The primary aim of this study is to compare infection-related hospitalizations and bacteremias between SPK and type 1 diabetic KTA patients after transplantation during 1-year and 5-year follow-up time. Our aim is also to characterize the site of infections, the risk factors for infection-related hospitalizations and bacteremias, as well as the impact of infection-related hospitalization and bacteremia on patient and graft survival in both groups. In addition, we compare overall patient and graft survival between SPK and KTA patients.

## Materials and Methods

We analyzed retrospectively all patients undergoing SPK for type 1 diabetes since the program was launched in Finland from March 2010 to December 2019. All transplantations were done in Helsinki University Hospital, the only transplant center in Finland. The control group consisted of patients with end-stage kidney disease secondary to type 1 diabetes who received KTA from a deceased donor in our institution during 2004–2013, which was before the routine implementation of the SPK program. The inclusion criteria for the controls were donor and recipient age < 60 and BMI < 30, the same age and weight limit used for SPK. This study had the approval of the Helsinki University Hospital institutional review board, and the Finnish Institute for Health and Welfare regarding the use of their administrative health data on hospitalizations in this study (THL/1877/5.05.00/2019).

All transplantations were ABO compatible and cytotoxic cross-match negative. For the SPK group, immunosuppression comprised tacrolimus, mycophenolate mofetil (MMF) and steroid. All SPK patients received induction with single-dose antithymocyte globulin (8 mg/kg) pre-transplantation. The post-transplantation trough level target for tacrolimus was 12–15 ug/L the first 14 days and 10–12 ug/L for days 15–90 after transplantation. From three to 12 months post-transplantation, the trough level target was 9–11 ug/L, from 12 to 24 months 8–10 ug/L, and thereafter 7–9 ug/L. Steroid was discontinued after 6 months unless donor-specified-antibodies existed, in which case methylprednisolone 2–4 mg remained as part of the immunosuppression. All transplantations were performed using enteric proximal jejunal exocrine drainage. For the KTA patients, baseline immunosuppression comprised primarily of cyclosporine combined with MMF and steroid. The cyclosporine trough level target was 170–200 ug/L for the first 3 months, from three to 6 months 160–190 ug/L, six to 12 months 100–120 ug/L, 12–24 months 80–120 ug/L, and thereafter 60–100 ug/L. Immunologically high-risk KTA patients received tacrolimus (trough level target 6–8 ug/mL for the first 3 months) and in selected cases induction therapy with basiliximab was administered. Steroid was usually discontinued after 1-year post-transplantation in KTA patients.

All SPK patients received perioperative antibiotic prophylaxis with piperacillin-tazobactam and ciprofloxacin in addition to anti-fungal prophylaxis with fluconazole or anidulafungin. These prophylaxis regimens were continued for three to 5 days postoperatively intravenously. In KTA patients, a single-dose of cefuroxime was administered during operation and another dose after operation. All patients received a 6-month prophylaxis for *Pneumocystis jirovecii* pneumonia with trimethoprim/sulfamethoxazole. Ureteral stent was removed 3–4 weeks after transplantation in both groups.

Six-month CMV prophylaxis with valganciclovir (900 mg once daily, or dose adjusted to renal function) was intended for all patients with CMV D+/R− constellation in both groups. This 6-month CMV prophylaxis protocol has been used since 2004 in our institution for these high-risk patients. Also, SPK patients with CMV R+ status received a 3-month valganciclovir prophylaxis since 2019, regardless of donor CMV status. KTA patients with other constellation besides D+/R− did not receive any prophylaxis. Patients without prophylaxis were monitored preemptively during their routine follow-up visits for DNAemia and antiviral treatment was initiated if the viral load exceeded 1,000 IU/mL. In viral loads lower than that, viremia was usually only monitored. In the case of treatment for acute rejection, CMV prophylaxis was given for one-to-three months depending on the used rejection treatment.

We analyzed all infections requiring hospital admission during 5 years after transplantation. Infections during the admission for transplantation were excluded due to higher risk of infections related to surgical complications in SPK. All SPK patients were followed at our institution and the hospitalizations gathered from the national transplant register and patient electronic medical records. In KTA patients, the infection related hospitalizations were gathered from the Finnish Care Register for Healthcare, which is a national administrative health registry maintained by the Finnish Institute for Health and Welfare, using ICD-10 codes A00–B99, J00–J99 and R50–R50.9 for primary diagnosis or as a secondary diagnosis when primary diagnosis was type 1 diabetes or diabetic nephropathy (N039*E10). Reporting of hospitalizations to the registry is mandatory by law. Additionally, in KTA patients, the follow-up data was also obtained from the national transplant register. The data were collected until 5 years from transplantation or until January 2022 in SPK patients who did not fulfil the 5-year follow-up time. Bacteremia was defined as presence of bacteria in the blood. Due to the lack of clinical information considering KTA patients, no further categorization was made.

The interval from transplantation to the first infection-related hospitalization was compared between SPK and KTA patients. The localizations of the infections were categorized as skin and soft tissue, gastrointestinal, pulmonary, pyelonephritis, unspecified, bacteremia, and CMV disease.

Statistical tests were performed using SPSS Version 28. For the comparison of study groups, 2-sided Mann-Whitney U-Test was used for continuous variables and chi-squared test for categorical variables. For the comparison of first infection-related hospitalizations or bacteremias between groups, Kaplan-Meier estimates were applied and censored for death or kidney graft loss which was defined as return to dialysis or death with functioning graft. Survival probabilities were also executed using Kaplan-Meier estimates. The SPK patients with initially functioning pancreas graft were included in the analysis. SPK patients who lost their pancreas graft, were still included in the SPK group after graft loss as they were exposed to the surgical procedure and the immunosuppression used in SPK patients. Pancreas graft failure was defined according to the definitions implemented in 2018 by the Organ Procurement and Transplantation Network (OPTN) including any of the following: recipient’s transplanted pancreas is removed, recipient reregisters for a pancreas transplant, recipient registers for an islet transplant after undergoing a pancreas transplant, recipient dies or recipient’s total insulin use is greater than or equal to 0.5 units/kg/day for 90 consecutive days. Cox regression models were used to study SPK as a risk factor for the first infection-related hospitalization and bacteremia after transplantation compared to KTA using only variables present at the time of transplantation. In the multivariable analysis SPK was adjusted with only recipient age and recipient sex since many of the other baseline characteristics are associated to SPK itself. Variables with *p* < 0.05 were considered statistically significant. All the infection-related hospitalizations and bacteremias during the follow-up was compared with incidence rate ratio since not all SPK patients fulfilled the 5-year follow-up. The effect of infection-related hospitalization or bacteremia on patient and kidney graft survival were studied with Cox’s regression using the first infection-related hospitalization or bacteremia as time-dependent variables, adjusted with patient’s age and sex.

## Results

Altogether 163 pancreas transplantations were performed between March 2010 and December 2019 in our institution. In total, 161 were SPK patients and two patients received pancreas after kidney transplantation. One patient with hyperacute pancreas graft rejection and immediate removal of the graft was excluded from the analyses resulting in 162 SPK patients included in the study. For the control group, 153 patients with end-stage kidney disease (ESKD) due to diabetic nephropathy who underwent KTA, met the inclusion criteria (recipient and donor BMI < 30 and age < 60) and were included in the study. The baseline characteristics of patients are shown in Table 1. The median follow-up time for SPK patients was 4.7 years (range 0.2–5.0, IQR 3.1–5.0) and for KTA patients 5.0 years (range 0.3–5.0, IQR 5.0–5.0). Altogether 84 patients of the SPK patients fulfilled the 5-year follow-up (unless died or lost their kidney graft) and 78 patients had a follow-up time varying from two-to-five years.

**TABLE 1:** Baseline characteristics of all the patients included in the study (*n* = 315).

Patient characteristics	SPK (*n* = 162)	KTA (*n* = 153)	*p*-value
Recipient age (years)	42.6 ± 8.1	45.3 ± 8.5	0.004
Recipient male sex (%)	108/163 (67)	101/153 (66)	0.91
Recipient BMI	24.2 ± 3.2	24.5 ± 3.1	0.39
Donor age (years)	38.5 ± 13.6	44.4 ± 13.1	<0.001
Donor male sex (%)	83/162 (51)	80/153 (52)	0.91
Donor BMI	23.6 ± 2.9	24.1 ± 2.9	0.09
Kidney cold ischemia time (min)	573 ± 124	1,298 ± 222	<0.001
Time in dialysis (months)	15.0 ± 11.3	29.5 ± 18.2	<0.001
Diabetes duration (years)	33.1 ± 8.2	33 ± 8.4	0.88
HLA-AB-mismatch	2.65 ± 0.9	1.65 ± 0.9	<0.001
HLA-DR-mismatch	1.49 ± 0.6	0.62 ± 0.5	<0.001
CMV D+/R− (%)	40/162 (25)	28/153 (18)	0.17
Hemodialysis before tx (%)	68/162 (42)	66/153 (43)	0.91
Peritoneal dialysis before tx (%)	88/162 (54)	85/153 (56)	0.91
Preemptive (%)	6/162 (4)	2/153 (1)	0.28
Kidney DGF (%)	18/162 (11)	40/153 (26)	<0.001
Rejection treatment (%)	53/162 (33)	26/153 (17)	0.002
Relaparotomy (%)	37/162 (23)	2/153 (1)	<0.001
Creatinine 1 year (mg/dL)^a^	1.3 ± 0.8	1.2 ± 0.4	0.48
Creatinine 5 years (mg/dL)^b^	1.2 ± 0.5	1.43 ± 0.63	0.04

All values presented as mean ± standard deviation unless otherwise noted. BMI, body max index; CMV, cytomegalovirus; D+/R−, pre-transplant donor seropositive/recipient seronegative to CMV; DGF, delayed graft function.

^a^ Data available in 156/162 SPK patients and 129/153 KTA patients.

^b^ Data available in 73/162 SPK patients and 122/153 KTA patients.

All SPK patients received tacrolimus-based immunosuppression and ATG induction. Among KTA patients, 128 (84%) were initially on cyclosporine and 25 (16%) on tacrolimus. Altogether 6 (4%) KTA patients received induction therapy with basiliximab and 147 (96%) did not receive any induction therapy. In KTA patients on cyclosporine, the mean trough level at three and 12 months was 169 ± SD 42 ug/L and 120 ± SD 32 ug/L, respectively. In SPK patients, mean tacrolimus trough level was 11.6 ± SD 3.4 ug/L at 3 months and 9.3 ± SD 2.4 ug/L at 12 months post-transplantation.

### Infection-Related Hospitalizations and Bacteremias After Transplantation

**FIGURE 1:**
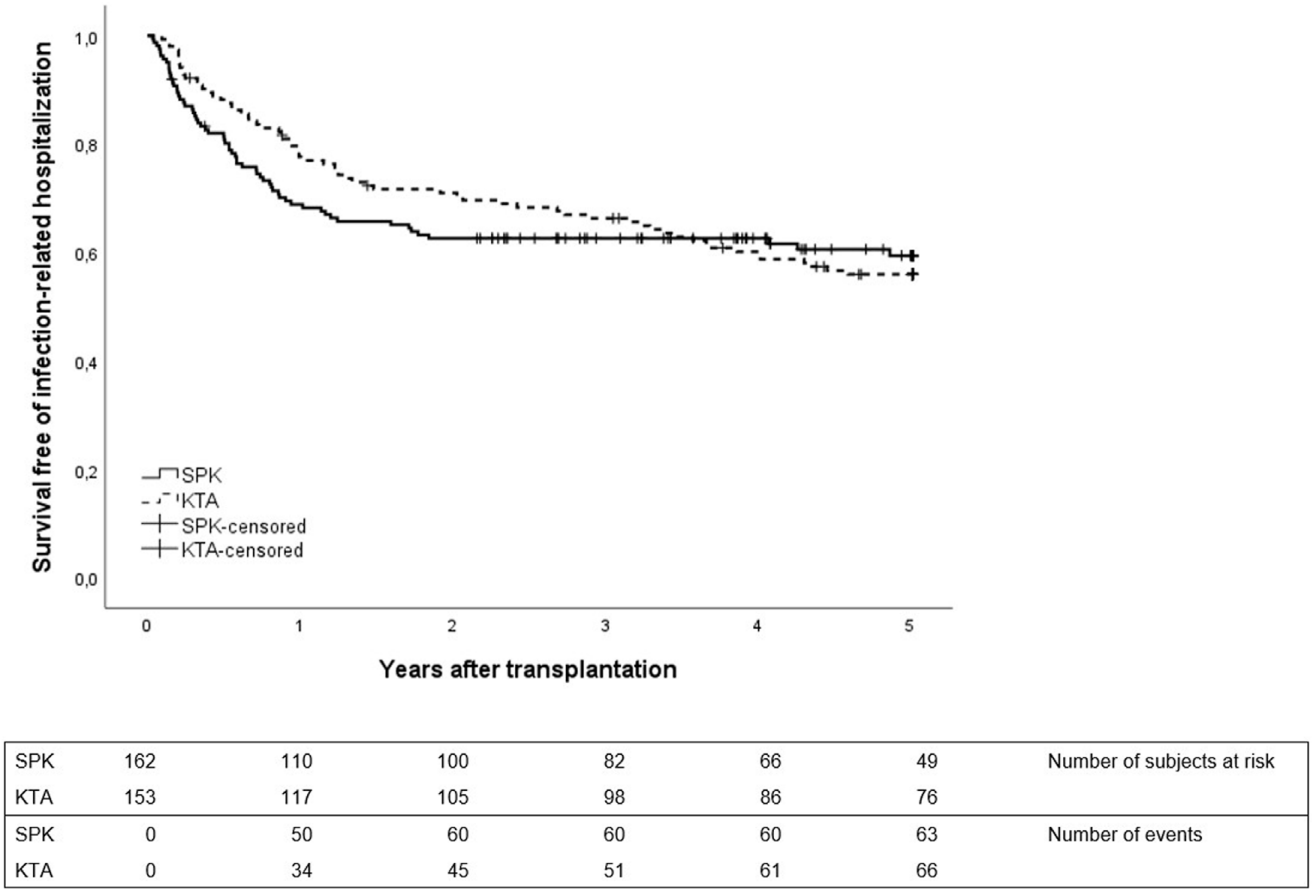
First infection-related hospitalization during 5 years after transplantation between SPK (simultaneous pancreas-kidney transplantation) and KTA (kidney transplantation alone) patients, *p* = 0.87.

The first infection-related hospitalization during 5-year follow-up time occurred earlier and mostly during the first year in SPK patients, whereas in KTA patients the first infection-related hospitalizations occurred later during follow-up (Figure 1). During the first year after transplantation, SPK patients had 0.54 infection-related hospitalizations/person year and KTA patients 0.31 infection-related hospitalizations/person year with an incidence rate ratio of 1.76 (95% CI 1.2211–2.5672, *p* = <0.001). During 5 years after transplantation, SPK patients and KTA patients had both 0.18 infection-related hospitalizations/person year, with an incidence rate ratio of 1.00 (95% CI 0.7971–1.3348, *p* = 0.81).

**FIGURE 2:**
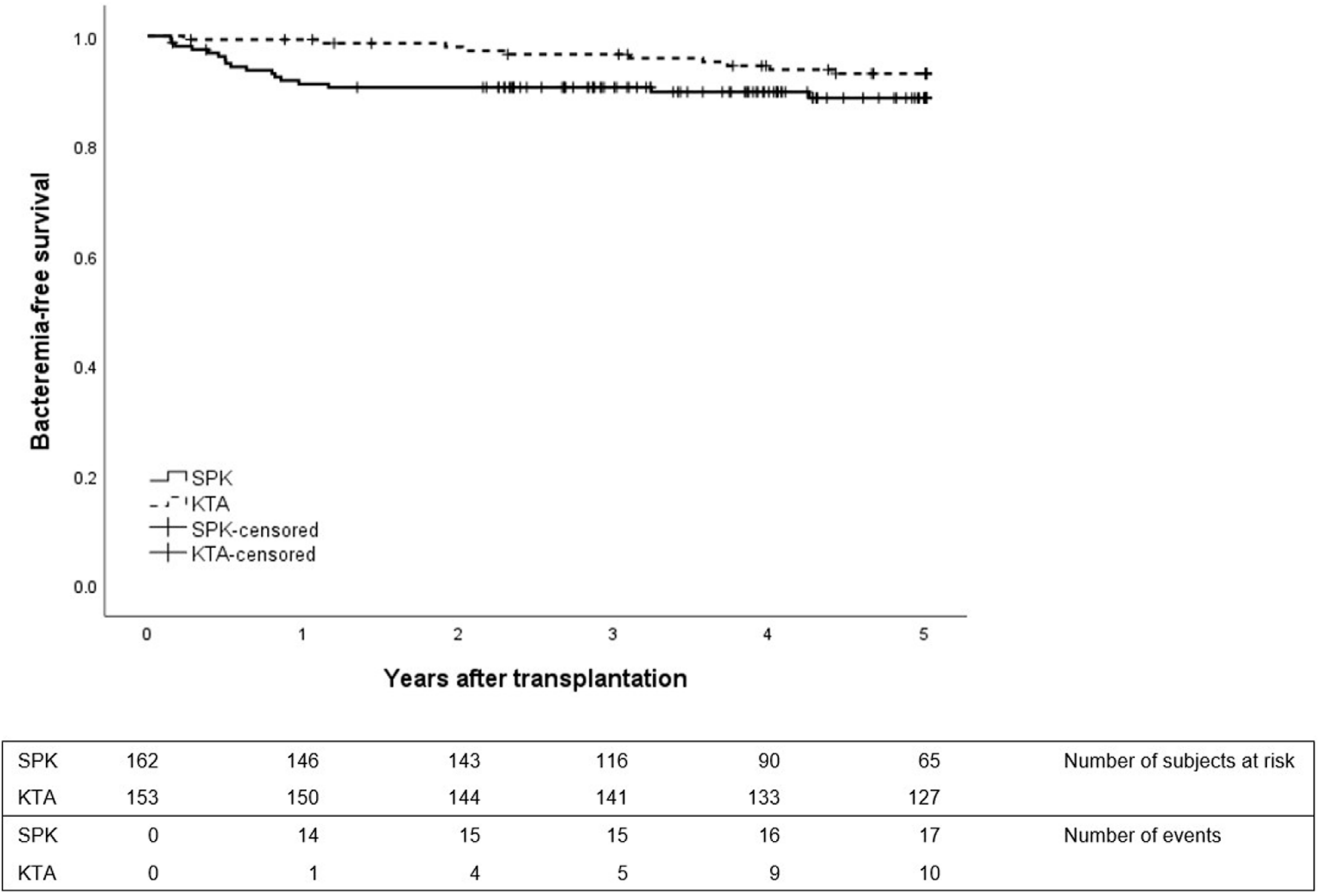
First bacteremia during 5 years after transplantation between SPK (simultaneous pancreas-kidney transplantation) and KTA (kidney transplantation alone) patients, *p* = 0.12.

Figure 2 depicts the first bacteremia during 5 years after transplantation between the groups. In SPK patients, the majority of bacteremias occurred during the first year while in KTA patients the first bacteremias occurred mainly after the first year and were divided more constantly for the following years. During the first year after transplantation, SPK patients had altogether 0.11 bacteremias/person year and KTA patients 0.01 bacteremias/person year with an incidence rate ratio of 17.12 (95% CI 2.704–713.40, *p* = <0.001). During 5 years after transplantation SPK patients had 0.034 bacteremias/person year and KTA patients 0.017 bacteremias/person year with an incidence rate ratio of 1.89 (95% CI 0.9052–4.059, *p* = 0.07).

### Site of Infections

The site of all the infections during the 5-year follow-up, including patients with multiple infection episodes, are shown in Table 2. In the SPK group, the most frequent cause of infection-related hospitalization were gastrointestinal infections (24 episodes, 20%) and bacteremias (22 episodes, 18%). From the gastrointestinal infections, the most common cause was *Clostridioides difficile* infection (nine episodes, 38%). One patient had persistent *Clostridioides difficile* enteritis and was admitted four times and finally treated with bezlotoxumab. Norovirus gastroenteritis accounted for 25% of the cases (six episodes). Three patients suffered from prolonged norovirus gastroenteritis and two of these patients received treatment with nitazoxanide. Altogether 25% of the gastrointestinal infections (six episodes) were of unknown etiology. Pulmonary infection in SPK (10 episodes, 8%) were mainly bacterial pneumonias with unknown pathogen. Urinary tract infections (UTI) in SPK patients (18 episodes, 15%) were mainly caused by *Escherichia Coli* and *Klebsiella pnemoniae*.

**TABLE 2:** Site of all the infection-related hospitalizations in SPK (simultaneous pancreas-kidney transplantation) and KTA (kidney transplantation alone) patients during 5 years after transplantation.

	SPK 120 episodes	KTA 143 episodes
Skin and soft tissue (%)	17/120 (14)	24/143 (17)
GI (%)	24/120 (20)	29/143 (20)
Pulmonary (%)	10/120 (8)	37/143 (26)
UTI (%)	18/120 (15)	9/143 (6)
Unspecified (%)	19/120 (16)	16/143 (11)
Bacteremia (%)	22/120 (18)	15/143 (11)
CMV (%)	10/120 (8)	13/143 (9)

GI, gastrointestinal; UTI, urinary tract infection; CMV, cytomegalovirus.

In the KTA group, pulmonary infections (37 episodes, 26%) were the most common cause for hospitalization and the majority were bacterial pneumonias and unspecified bronchitises. Gastrointestinal infections were the second most common site of infection (29 episodes, 20%) with the majority of unknown etiology. Hospitalization due to UTI was only 6% (9 episodes), with *Escherichia Coli* the main pathogen.

All the pathogens causing bacteremia are listed in Table 3, in both groups, the most common pathogens causing bacteremia were *Staphylococcus aureus* and *Escherichia coli*.

**TABLE 3:** All pathogens for bacteremia in SPK and KTA patients during the 5-year follow-up.

SPK (22 episodes)	KTA (15 episodes)
*Escherichia Coli 8*	*Escherichia Coli 5*
*Staphylococcus Aureus 6*	*Staphylococcus Aureus 4*
*Enterococcus Cloacae*	*Pseudomonas 2*
*Klebsiella Pneumoniae*	*Klebsiella Pneumoniae 2*
*Staphylococcus heamolyticus*	*Unspecified pathogen 2*
*Pseudomonas*	
*Candida Albicans*	
*Enterobacter species*	
*Enterococcus feacium*	
*Candica Glabrata*	

### The Outcome in SPK and KTA With Infection Related Hospitalization or Bacteremia

Infection-related hospitalization was not related to worse patient or kidney graft survival compared to patients without infection-related hospitalization in 5-year follow-up in either group (Supplementary Figures S1A–D). However, in both groups, bacteremia was associated with both inferior kidney graft and patient survival (Supplementary Figures S2A–D).

### Risk Factor Analysis

The results of univariable and multivariable analyses of risk factors associated with infection-hospitalization and bacteremia during the first year after transplantation are shown in Tables 4, 5, respectively. In the univariable analysis for infection-related hospitalization, no significant risk factors were found. When adjusted with recipient age and sex in the multivariable model, SPK was identified as a risk factor for infection-related hospitalization during the first year after transplantation. In addition, SPK was a risk factor for bacteremia in both univariable and multivariable models during the first year after transplantation.

**TABLE 4:** Hazard Ratios (HR) with 95% confidence intervals by Cox’s regression of the risk factors for infection-related hospitalization during the first year after transplantation.

	Univariable (95 % CI)	Multivariable (95 % CI)
SPK vs. KTA	1.5 (1.0–2.4), *p* = 0.06	1.6 (1.0–2.5), *p* = 0.04
Recipient age	1.0 (1.0–1.0), *p* = 0.34	1.0 (1.0–1.0), *p* = 0.2
Recipient male sex	1.0 (0.6–1.5), *p* = 0.90	0.9 (0.6–1.5), *p* = 0.77
Recipient BMI	1.0 (0.9–1.1), *p* = 0.61	
Donor age	1.0 (1.0–1.0), *p* = 0.28	
Donor male sex	0.9 (0.6–1.3), *p* = 0.47	
Time in dialysis	1.0 (1.0–1.0), *p* = 0.14	
Diabetes duration	1.0 (1.0–1.0), *p* = 0.98	
DGF (kidney)	1.6 (1.0–2.7), *p* = 0.06	

BMI, body max index; DGF, delayed graft function; SPK simultaneous pancreas-kidney transplantation; KTA, kidney transplantation alone.

**TABLE 5:** Hazard Ratios (HR) with 95% confidence intervals by Cox’s regression of the risk factors for bacteremia during the first year after transplantation.

	Univariable (95 % CI)	Multivariable (95 % CI)
SPK vs. KTA	13.8 (1.8–104.7), *p* = 0.01	16.3 (2.1–125.1), *p* = 0.01
Recipient age	1.0 (1.0–1.1), *p* = 0.32	1.1 (1.0–1.1), *p* = 0.1
Recipient male sex	0.6 (0.2–1.6), *p* = 0.27	0.5 (0.2–1.4), *p* = 0.2
Recipient BMI	1.0 (0.8–1.2), *p* = 0.92	
Donor age	1.0 (1.0–1.1), *p* = 0.18	
Donor male sex	1.1 (0.4–2.9), *p* = 0.91	
Time in dialysis	1.0 (0.9–1.0), *p* = 0.09	
Diabetes duration	1.1 (1.0–1.1), *p* = 0.11	
DGF (kidney)	1.1 (0.3–3.9), *p* = 0.90	

BMI, body max index; DGF, delayed graft function; SPK simultaneous pancreas-kidney transplantation; KTA, kidney transplantation alone.

In the 5-year risk analysis for infection-related hospitalization and bacteremia, SPK was not a risk factor in the univariable analysis or when adjusted with recipient age and sex. Donor age was found to be a risk factor for bacteremia in the univariable analysis (Supplementary Tables S1, S2).

### Mortality and Graft Survival

Altogether 7/162 (3.7%) SPK patients died during follow-up with functioning grafts. Three of these deaths were considered infection-related, one patient died from pulmonary embolism 8 months post-transplantation after being treated for bacteremia, one patient died from complicated atypical mycobacterial infection combined with pancreatitis of the patient’s native pancreas 4 months after transplantation, and one patient died due to Fournier’s gangrene and septic shock 10 months after transplantation.

In addition, altogether six patients experienced pancreas graft failure during follow-up and the death-censored 5-year pancreas graft survival was 96.3% Five pancreas grafts were removed during follow-up and four of these were removed during the first 3 months due to persistent intra-abdominal fungal infections. One patient with severe leukopenia had recurrent infections and was diagnosed with necrotic ulcer in the bowel. This progressed into septic fungal infection and pancreas graft had to be removed 9 months after transplantation. In addition, one patient had pancreas graft failure without known reason 32 months after transplantation and returned to full-dose insulin treatment.

Furthermore, four patients had deteriorated pancreas function and required insulin treatment despite detectable C-peptide concentration. Also, 13 patients developed insulin-resistance during follow-up and required oral hypoglycemic therapy. In patients with SPK, all kidney grafts were functioning at the end of the follow-up.

In the KTA group, 12/153 (8.5%) patients died during the 5-year follow-up. Only 1/13 of the deaths was considered as infection-related, a patient who died from urosepsis 44 months after transplantation. In addition, seven patients returned to dialysis during follow-up. The earliest return to dialysis occurred 2 years 4 months after transplantation.

**FIGURE 3:**
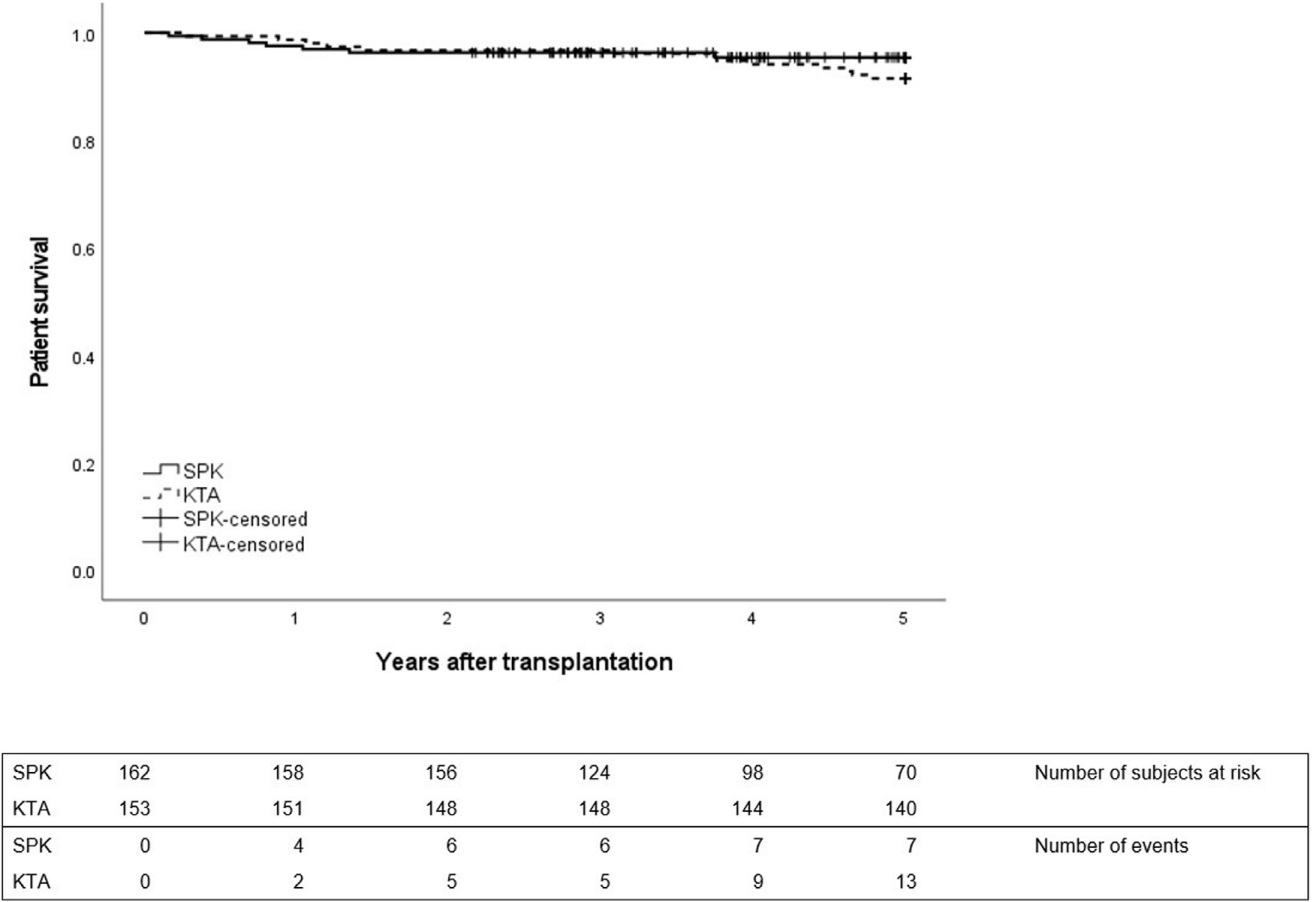
Patient survival during 5 years after transplantation between SPK (simultaneous pancreas-kidney transplantation) and KTA (kidney transplantation alone) patients, *p* = 0.32.

**FIGURE 4:**
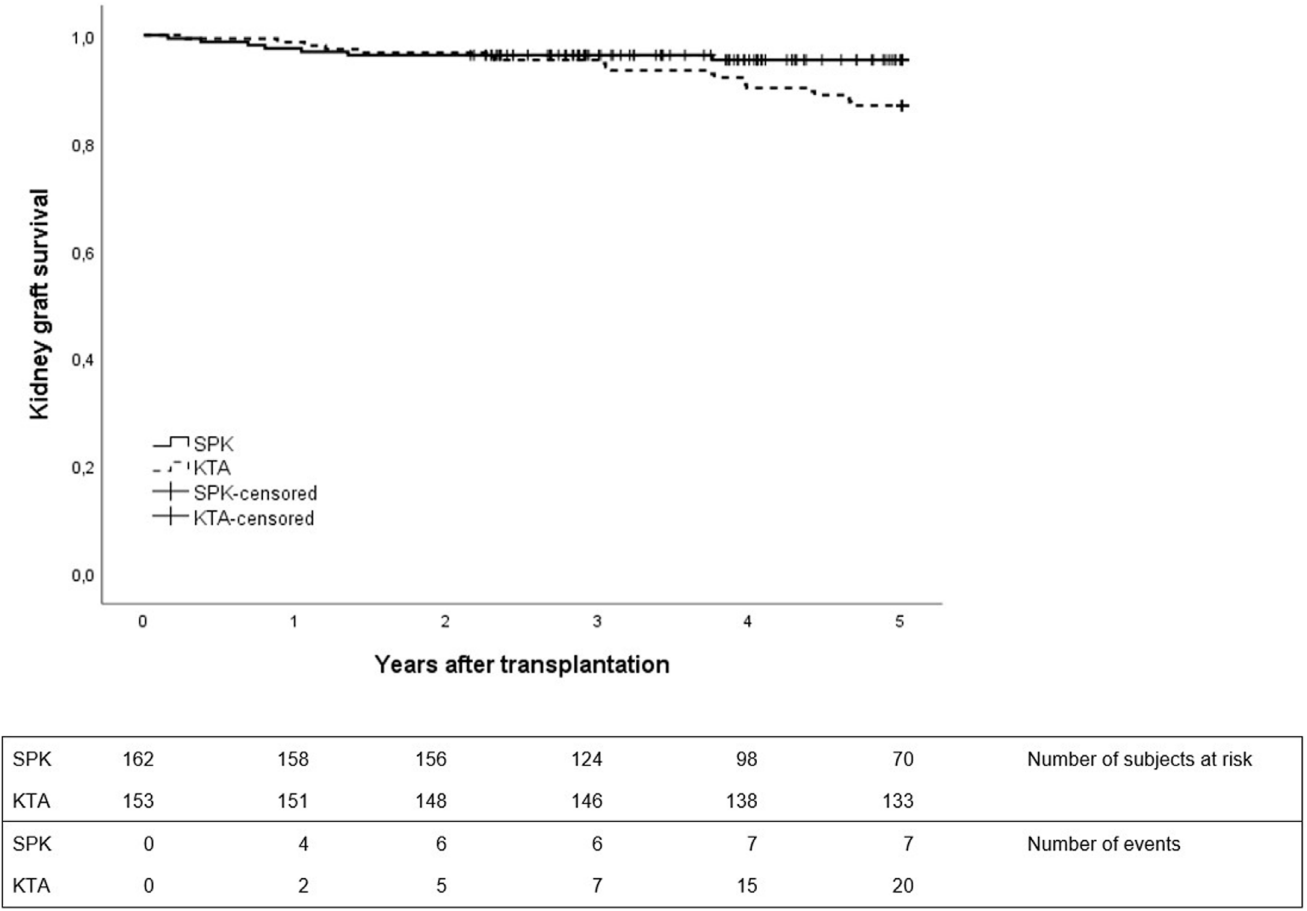
Kidney graft survival during 5 years after transplantation between SPK (simultaneous pancreas-kidney transplantation) and KTA (kidney transplantation alone) patients, *p* = 0.04.

No differences were observed in patient survival between groups during 1- or 5-year follow-up between SPK and KTA patients (Figure 3). However, at the end of the 5-year follow-up, kidney graft survival was lower in KTA patients compared to SPK patients (Figure 4).

## Discussion

Our study showed that the risk of infection-related hospitalizations and bacteremias concentrates especially to the first post-transplant year after SPK and is higher compared to KTA patients with type 1 diabetes. However, during longer follow-up the risk of infections declines in SPK patients whereas in KTA patients, the infection-related hospitalizations, mainly due to community acquired infections, become increasingly common.

Previous studies have indicated that the incidence of infections has been high during the early post-transplant phase in SPK patients, but the risk of infections seems to decline in the long run [20, 21]. Similarly, our study showed that majority of first infections requiring hospitalization occurred during the first year after transplantation in SPK patients. In a study comparing the rate of sepsis in SPK and KTA patients, SPK patients showed higher incidence and an earlier onset of sepsis compared to KTA patients [22], which also is in line with our results even though we excluded early infections during the primary admission and focused on the later posttransplant course. Of note, in our study we specifically compared SPK patients only with KTA patients with type 1 diabetes, whereas previous studies have used KTA patients from all ESKD etiologies as controls.

When assessing the site of infections, gastrointestinal infections were the most common reasons for hospitalization in SPK patients, and the second most common reason in the KTA group. In SPK patients, the majority of identified pathogens were *Clostridioides difficile* and norovirus. Overall, the high risk of gastrointestinal infections in SPK patients may be related to surgery involving the bowel, and *Clostridioides difficile* infections to ATG induction therapy and longer prophylactic antibiotic treatment. In previous study, age older than 55 years, transplant other than kidney transplantation alone, and ATG induction were associated with higher risk of *Clostridioides difficile* associated diarrhea in SOT patients [23]. Pneumonia or infection of the upper respiratory tract was the most common reason for hospitalization in KTA patients accounting for 26% of all hospitalizations. In a large multicenter study in SOT recipients, pneumonia was similarly a frequent complication after transplantation, and among renal transplant recipients over half of the cases occurred later (>6 months) after transplantation [24]. The low incidence of infection-related hospitalizations due to UTIs in our study is probably due to the fact that bacteremias that were derived from the urinary tract were classified as bacteremias not as UTIs. In addition, as this study focused only on the infection-related hospitalizations, it does not provide information about the overall risk of UTIs after transplantation.

In risk factor analysis, SPK was found to be a risk factor for infection-related hospitalizations and bacteremia during the first year after transplantation but no longer during the 5-year follow-up compared to KTA patients. This suggests that stronger immunosuppression and the use of lymphocyte-depleting agents predisposes SPK patients to infections especially during the first year after transplantation. In addition, the higher rate of relaparotomy and rejection after SPK is a possible explanation for the higher infection risk. Female sex was a near-significant risk factor for bacteremia, the association of recipient female sex and bacteremia has been shown in a previous large cohort study in kidney transplant recipients and is most likely relates to the increased risk for urinary tract infections [25].

According to the U.S. Renal Data System Annual Data Report, sepsis was one of the most commonly known cause of mortality among kidney transplant recipients, in addition to cardiovascular causes and malignancies [26]. In our study, bacteremia was associated with inferior kidney graft and patient survival in both groups.

When comparing overall patient and kidney graft survival between groups, no difference was detected in patient survival. However, kidney graft survival was inferior in KTA group during 5-year follow-up, as none of the SPK patients alive at the end of the follow-up lost their kidney transplant. These excellent results of kidney graft outcome in SPK patients were also demonstrated by the recent OPTN/SRTR Annual Data Report on pancreas transplantation [27]. In our patients, five pancreas grafts had to be removed and four of these graft removals were due to persistent intra-abdominal fungal infections, emphasizing the high risk of graft loss related to fungal infections in SOT patients [28].

Our study had some limitations of note and the most important limitation to our study is the difference in immunosuppression between the groups. All SPK patients received lymphocyte-depleting induction and tacrolimus-based immunosuppression which probably explains the higher risk of infections during the first post-transplant year. Despite this, the risk of infections seems to be similar during the first 5 years suggesting that improved glycemic control in SPK patients could protect SPK patients from infectious risks. Second, this was a single center study as our center is the only transplant center in Finland, and results may not be comparable to other populations. Third, KTA patients were selected by the recipient and donor BMI and age criteria, similar to the criteria used for SPK patients in our center, from a time period of 2004–2009 when SPK was not performed in Finland, or during the early years of SPK transplantation in 2010–2013, when the activity was very low. Our baseline assumption was that during later years, these patients could have been considered for SPK. Baseline characteristics were relatively similar between the groups regarding diabetes duration and BMI, although SPK patients were slightly younger and had shorter waiting time to transplantation, and therefore shorter exposure to pretransplant dialysis treatment. There were also differences in HLA mismatch, cold ischemia time, and prophylactic antibiotic regimens between the groups, resulting in possible bias in our findings. We acknowledge that there might be also other unmeasured factors, associated with either the type of transplantation, or the era of transplantation, that could confound our findings. Also, the extension of the CMV prophylaxis criteria in SPK patients in 2019 that may have decreased the hospitalizations caused by CMV. In addition, not all the SPK patients fulfilled the 5-year follow-up, limiting our possibilities to compare infection-related hospitalizations during the whole study period.

In conclusion, simultaneous pancreas-kidney transplantation (SPK) patients are at greater risk for infection-related hospitalizations and bacteremias compared to kidney transplantation alone (KTA) patients with type 1 diabetes during the first year after transplantation, which may be associated with the use of stronger immunosuppression and lymphocyte-depleting induction in simultaneous pancreas-kidney transplantation, and this should be taken account during pretransplant evaluation for candidacy. However, during longer follow-up, the risk of infection-related hospitalizations was similar between SPK patients and KTA patients, suggesting that the relative risk of infections after the first posttransplant year is lower among SPK patients.

## Data Availability

The raw data supporting the conclusion of this article will be made available by the authors, without undue reservation.

## Abbreviations

CMV, cytomegalovirus; D+/R−, donor cytomegalovirus positive, recipient negative; DGF, delayed graft function; GI, gastrointestinal; KTA, kidney transplantation alone; MMF, mycophenolate mofetil; SOT, solid organ transplantation; SPK, simultaneous pancreas-kidney transplantation; UTI, urinary tract infection.
