# Supplementary material for: Infection-Related Hospitalizations After Simultaneous Pancreas-Kidney Transplantation Compared to Kidney Transplantation Alone
Source: Transpl Int. 2024 Feb 20;37:12235. doi: 10.3389/ti.2024.12235 (PMC10912468; doi:10.3389/ti.2024.12235)
Supplement: Supplementary file 1 [file DataSheet1.docx]

Supplement, Figure 1a. Risk of death associated with infection-related hospitalization among SPK (simultaneous pancreas-kidney tranplantation) patients during five years after transplantation.


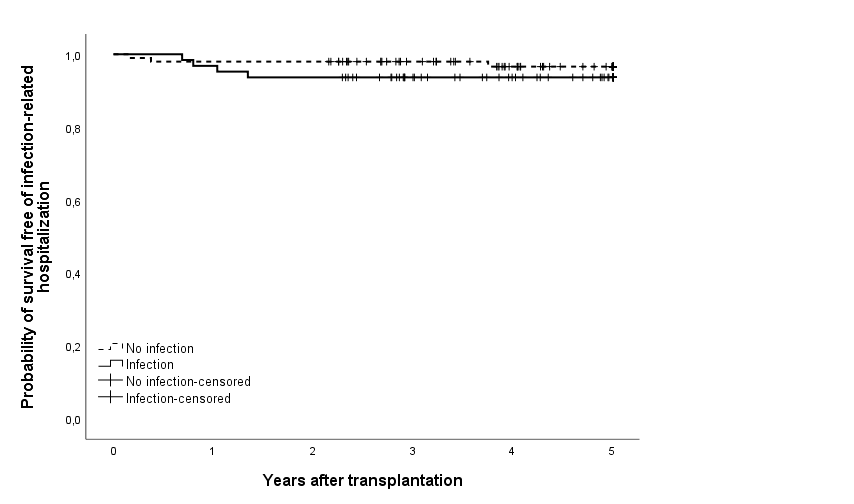


|  | Univariable (CI 95%) | Multivariable (CI 95%) |
| --- | --- | --- |
| Infection-related hospitalization  (time-dependent) | 2.4 (0.5-11.4), p=0.26 | 2.7 (0.55-13.7), p=0.22 |
| Recipient age | **1.1 (1.0-1.3), p=0.04** | 1.1 (1.0-1.2), p=0.07 |
| Recipient male sex | 38.5 (0.1-27619.4), p=0.28 | 325629.1 (0.0-1.7e266), p=0.97 |

Supplement, Figure 1b. Risk of kidney graft failure associated with infection-related hospitalization among SPK (simultaneous pancreas-kidney tranplantation) patients during five years after transplantation.


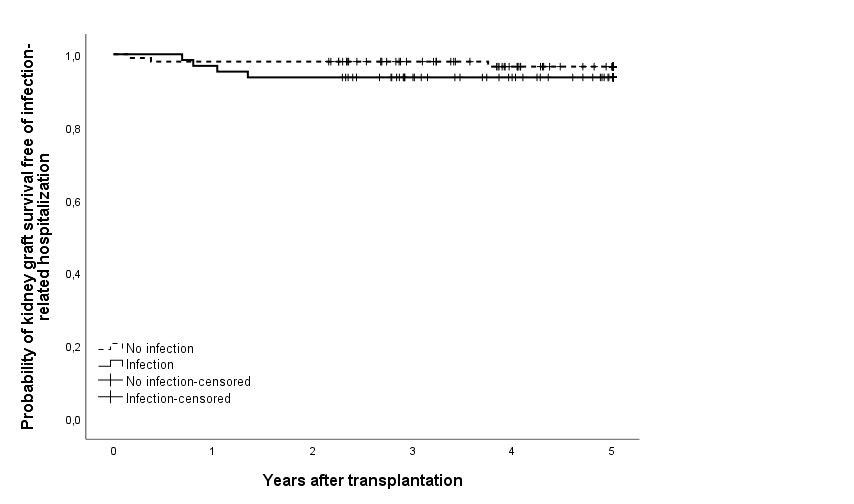


|  | Univariable (CI 95%) | Multivariable (CI 95%) |
| --- | --- | --- |
| Infection-related hospitalization  (time-dependent) | 2.4 (0.5-11.4), p=0.26 | 2.7 (0.55-13.7), p=0.22 |
| Recipient age | **1.1 (1.0-1.3), p=0.04** | 1.1 (1.0-1.2), p=0.07 |
| Recipient male sex | 38.5 (0.1-27619.4), p=0.28 | 325629.1 (0.0-1.7e266), p=0.97 |

Supplement, Figure 1c. Risk of death associated with infection-related hospitalization among KTA (kidney transplantation alone) patients during five years after transplantation


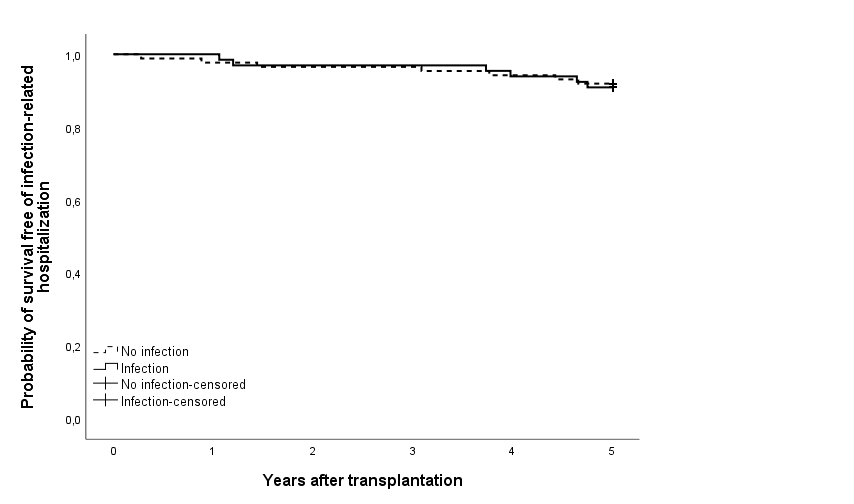


|  | Univariable (CI 95%) | Multivariable (CI 95%) |
| --- | --- | --- |
| Infection-related hospitalization  (time-dependent) | 1.9 (0.6-5.8), p=0.28 | 1.8 (0.6-5.5), p=0.32 |
| Recipient age | 1.0 (1.0-1.1), p=0.3 | 1.0 (1.0-1.1), p=0.37 |
| Recipient male sex | 1.8 (0.5-6.4), p=0.4 | 1.6 (0.4-5.8), p=0.48 |

Supplement, Figure 1d. Risk of kidney graft failure associated with infection-related hospitalization among KTA (kidney transplantation alone) patients during five years after transplantation


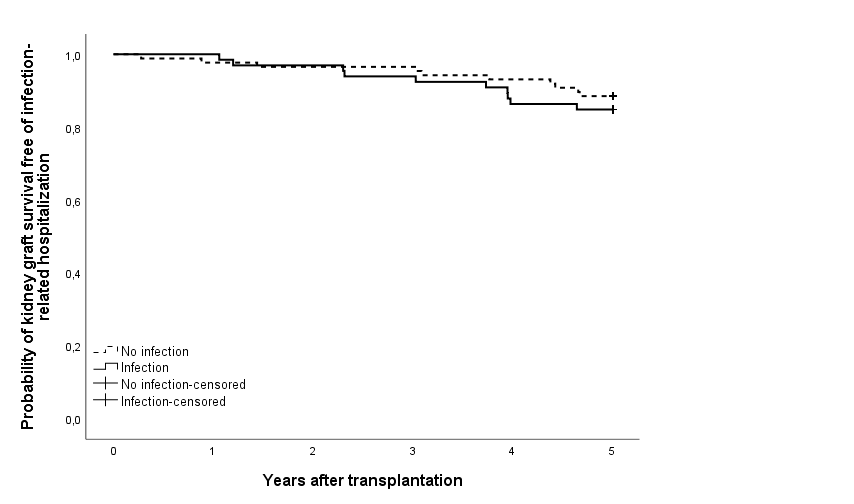


|  | Univariable (CI 95%) | Multivariable (CI 95%) |
| --- | --- | --- |
| Infection-related hospitalization (time-dependent) | 2.2 (0.9-5.3), p=0.1 | 2.2 (0.9-5.3), p=0.1 |
| Recipient age | 1.0 (1.0-1.1), p=0.92 | 1.0 (0.9-1.1), p=0.79 |
| Recipient male sex | 1.2 (0.5-3.1), p=0.72 | 1.1 (0.4-3.0), p=0.81 |

Supplement, Figure 2a. Risk of death associated with bacteremia among SPK (simultaneous pancreas-kidney tranplantation) patients during five years after transplantation.


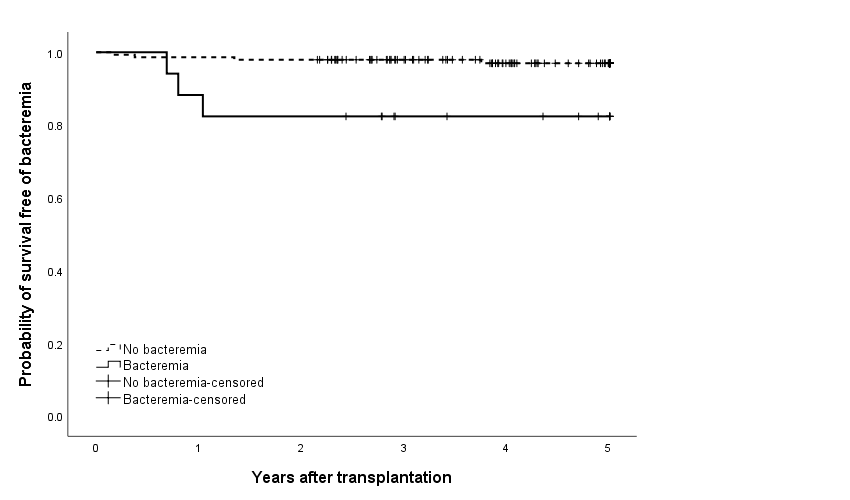


|  | Univariable (CI 95%) | Multivariable (CI 95%) |
| --- | --- | --- |
| Bacteremia (time-dependent) | **7.5 (1.4-40.7), p=0.02** | **9.3 (1.5-58.0), p=0.02** |
| Recipient age | **1.0 (1.0-1.3), p=0.04** | 1.1 (1.0-1.2), p=0.11 |
| Recipient male sex | 38.5 (0.05-27619.4), p=0.28 | 475591.2 (0.0-3.9 e 288), p=0.97 |

Supplement, Figure 2b. Risk of kidney graft failure associated with bacteremia among SPK (simultaneous pancreas-kidney tranplantation) patients during five years after transplantation.


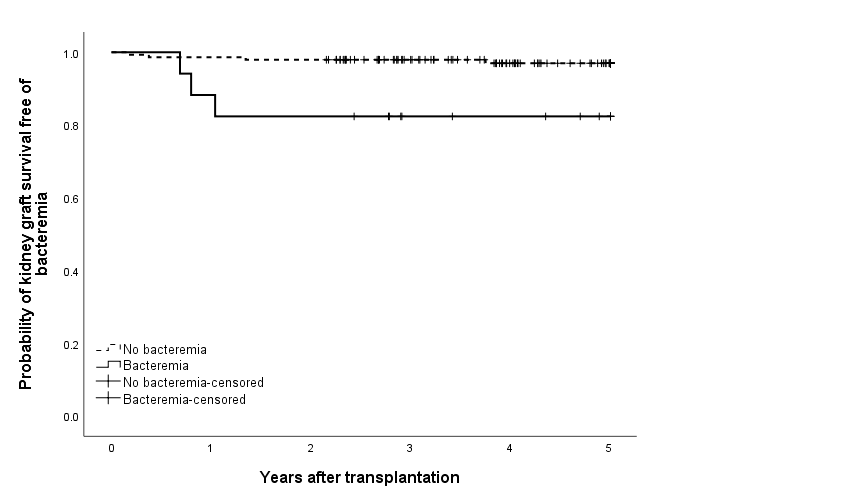


|  | Univariable (CI 95%) | Multivariable (CI 95%) |
| --- | --- | --- |
| Bacteremia (time-dependent) | **7.5 (1.4-40.7), p=0.02** | **9.3 (1.5-58.0), p=0.02** |
| Recipient age | **1.0 (1.0-1.3), p=0.04** | 1.1 (1.0-1.2), p=0.11 |
| Recipient male sex | 38.5 (0.05-27619.4), p=0.28 | 475591.2 (0.0-3.9 e 288), p=0.97 |

Supplement, Figure 2c. Risk of death associated with bacteremia among KTA (kidney transplantation alone) patients during five years after transplantation


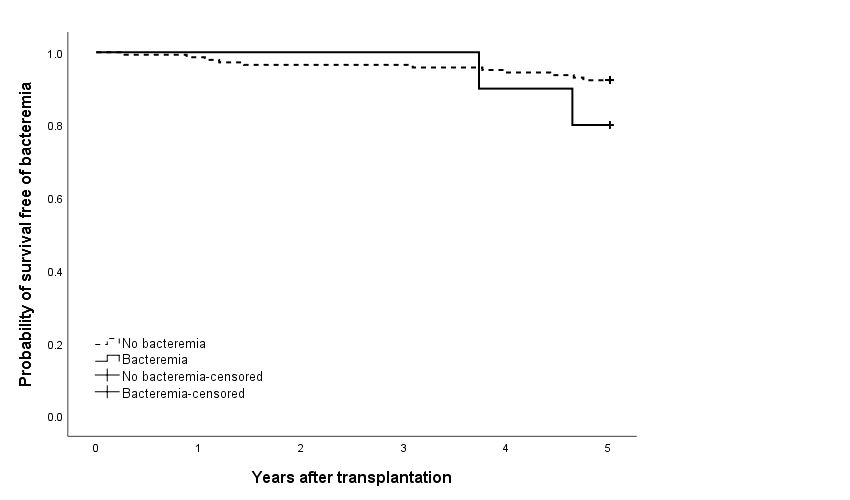


|  | Univariable (CI 95%) | Multivariable (CI 95%) |
| --- | --- | --- |
| Bacteremia (time-dependent) | **5.1 (1.1-24.3), p=0.04** | **5.6 (1.2-26.3), p=0.03** |
| Recipient age | 1.0 (1.0-1.1), p=0.3 | 1.0 (1.0-1.1), p=0.32 |
| Recipient male sex | 1.8 (0.5-6.4), p=0.4 | 1.7 (0.5-6.2), p=0.44 |

Supplement, Figure 2d. Risk of kidney graft failure associated with bacteremia among KTA (kidney transplantation alone) patients during five years after transplantation.


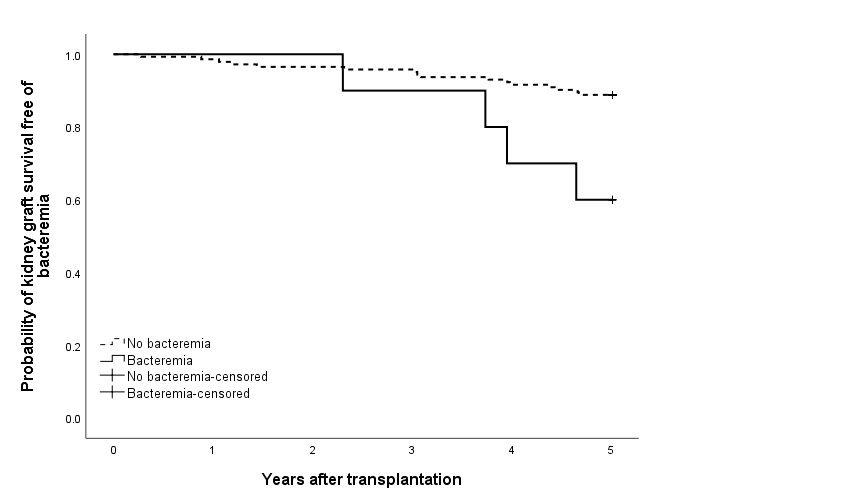


|  | Univariable (CI 95%) | Multivariable (CI 95%) |
| --- | --- | --- |
| Bacteremia (time-dependent) | **8.0 (2.6-24.6), p=<0.001** | **8.1 (2.6-25.0), p=<0.001** |
| Recipient age | 1.0 (1.0-1.1), p=0.92 | 1.0 (1.0-1.1), p=0.94 |
| Recipient male sex | 1.2 (0.5-3.1), p=0.72 | 1.2 (0.5-3.2), p=0.71 |

Supplement, Table 1. Hazard Ratios (HR) with 95% confidence intervals by Cox’s regression of the risk factors for infection-related hospitalization during five years after transplantation

|  | Univariable (95 % CI) | Multivariable (95% CI) |
| --- | --- | --- |
| SPK vs KTA | 1.0 (0.7-1.5), p= 0.87 | 1.1 (0.8-1.6), p=0.61 |
| Recipient age | 1.0 (1.0-1.0) p= 0.14 | 1.0 (1.0-1.0), p=0.11 |
| Recipient male sex | 0.8 (0.6-1.2) p=0.28 | 0.8 (0.6-1.2), p=0.22 |
| Recipient BMI | 1.0 (0.9-1.0) p= 0.58 |  |
| Donor age | 1.0 (1.0-1.0) p=0.12 |  |
| Donor male sex | 0.9 (0.7-1.3), p=0.7 |  |
| Time in dialysis | 1.0 (1.0-1.0) p=10 |  |
| Diabetes duration | 1.0 (1.0- 1.0) p= 0.79 |  |
| DGF (kidney) | 1.48 (1.0-2.2), p=0.06 |  |

CI, confidence interval; BMI, body max index; DGF, delayed graft function; SPK simultaneous pancreas-kidney transplantation; KTA, kidney transplantation alone

Supplement, Table 2. Hazard Ratios (HR) with 95% confidence intervals by Cox’s regression of the risk factors for bacteremia during five years after transplantation

|  | Univariable (95 % CI) | Multivariable (95 % CI) |
| --- | --- | --- |
| SPK vs KTA | 1.9 (0.9-4.1), p= 0.12 | 2.0 (0.9-4.5), p=0.08 |
| Recipient age | 1.0 (1.0-1.1), p=0.54 | 1.0 (1.0-1.1), p=0.29 |
| Recipient male sex | 0.5 (0.3-1.1), p=0.1 | 0.5 (0.2-1.1), p=0.08 |
| Recipient BMI | 1.0 (0.9-1.1), p=0.54 |  |
| Donor age | **1.0 (1.0-1.1), p=0.04** |  |
| Donor male sex | 1.0 (0.5-2.1), p=0.95 |  |
| Time in dialysis | 1.0 (1.0-1.0), p=0.20 |  |
| Diabetes duration | 1.0 (1.0-1.1), p= 0.29 |  |
| DGF (kidney) | 0.8 (0.3–2.6). p=0.65 |  |

CI, confidence interval; BMI, body max index; DGF, delayed graft function; SPK simultaneous pancreas-kidney transplantation; KTA, kidney transplantation alone
